# Extracellular ATP Limits Homeostatic T Cell Migration Within Lymph Nodes

**DOI:** 10.3389/fimmu.2021.786595

**Published:** 2021-12-22

**Authors:** Daichi Kobayashi, Yuki Sugiura, Eiji Umemoto, Akira Takeda, Hisashi Ueta, Haruko Hayasaka, Shinsuke Matsuzaki, Tomoya Katakai, Makoto Suematsu, Itaru Hamachi, Gennady G. Yegutkin, Marko Salmi, Sirpa Jalkanen, Masayuki Miyasaka

**Affiliations:** ^1^ Department of Immunology, Niigata University Graduate School of Medical and Dental Sciences, Niigata, Japan; ^2^ Department of Pharmacology, Wakayama Medical University, Wakayama, Japan; ^3^ Department of Biochemistry, Keio University School of Medicine, Tokyo, Japan; ^4^ Laboratory of Microbiology and Immunology, University of Shizuoka, Shizuoka, Japan; ^5^ MediCity Research Laboratory, University of Turku, Turku, Finland; ^6^ Department of Anatomy, School of Medicine, Dokkyo Medical University, Tochigi, Japan; ^7^ Laboratory of Immune Molecular Function, Faculty of Science and Engineering, Kindai University, Higashi-Osaka, Japan; ^8^ Department of Radiological Sciences, Morinomiya University of Medical Sciences, Osaka, Japan; ^9^ Department of Synthetic Chemistry and Biological Chemistry, Graduate School of Engineering, Kyoto University, Kyoto, Japan; ^10^ Institute of Biomedicine, University of Turku, Turku, Finland; ^11^ Department of Microbiology and Immunology, Osaka University Graduate School of Medicine, Suita, Japan; ^12^ World Premier International (WPI) Immunology Frontier Research Center, Osaka University, Suita, Japan

**Keywords:** adenosine 5’-triphosphate (ATP), T cells, chemokines, cell migration, lymph nodes (LNs)

## Abstract

Whereas adenosine 5’-triphosphate (ATP) is the major energy source in cells, extracellular ATP (eATP) released from activated/damaged cells is widely thought to represent a potent damage-associated molecular pattern that promotes inflammatory responses. Here, we provide suggestive evidence that eATP is constitutively produced in the uninflamed lymph node (LN) paracortex by naïve T cells responding to C-C chemokine receptor type 7 (CCR7) ligand chemokines. Consistently, eATP was markedly reduced in naïve T cell-depleted LNs, including those of nude mice, CCR7-deficient mice, and mice subjected to the interruption of the afferent lymphatics in local LNs. Stimulation with a CCR7 ligand chemokine, CCL19, induced ATP release from LN cells, which inhibited CCR7-dependent lymphocyte migration *in vitro* by a mechanism dependent on the purinoreceptor P2X7 (P2X7R), and P2X7R inhibition enhanced T cell retention in LNs *in vivo*. These results collectively indicate that paracortical eATP is produced by naïve T cells in response to constitutively expressed chemokines, and that eATP negatively regulates CCR7-mediated lymphocyte migration within LNs *via* a specific subtype of ATP receptor, demonstrating its fine-tuning role in homeostatic cell migration within LNs.

## Introduction

ATP serves as the major energy source in cells and once released from the cell, it provides strong inflammatory signals even at low concentrations, whereas its metabolite, adenosine, provides anti-inflammatory signals, through specific purinergic receptors expressed on the surface of cells (1–4). Extracellular ATP (eATP) thus represents a potent danger signal that alerts the immune system. Supporting this idea, the administration of hydrolysis-resistant ATP analogs enhanced antigen-induced cellular (5, 6) and humoral (7) immune responses, leading to the proposal that eATP functions as an endogenous adjuvant that enhances immune responses. In addition, isolated T cells were shown to produce eATP subsequent to antigenic stimulation (8–12), which resulted in the autocrine stimulation of specific ATP receptors expressed on T cells, leading to enhanced T cell activation (8). These results further strengthen the idea that ATP is a proinflammatory molecule that enhances immune responses upon release from the cell.

Therefore, it is thought that lymphocytes are exposed to negligible concentrations of eATP within lymphoid tissues under physiological conditions and that eATP appears when lymphocytes are stimulated by antigens (8, 13). Nevertheless, evidence indicates that all tissues and cell types regularly release ATP and that eATP is present at biologically significant levels in tissues under non-inflamed conditions (14–16). To the best of our knowledge, no experimental study has verified the presence or absence of eATP in uninflamed immunological tissues or its role in homeostasis.

*In vivo*, purine nucleotides are rapidly degraded by the combined actions of various ectonucleotidases, including CD39/ectonucleoside triphosphate diphosphohydrolase-1 (ENTPD-1) that converts ATP and adenosine 5’-diphosphate (ADP) to adenosine 5′-monophosphate (AMP), and CD73/ecto-5′-nucleotidase that converts AMP to adenosine (17). Of note, CD39 and CD73 ectonucleotidases were highly expressed in an enzymatically active form in the apparently uninflamed lymph nodes (LNs) of mice and humans (18), suggesting that although eATP arises under inflammatory conditions, it is constitutively but possibly transiently present in resting LNs.

The *in situ* demonstration of eATP within a tissue is technically challenging for several reasons: ATP is rapidly released from dying or stressed cells during tissue manipulation, and it is rapidly hydrolyzed during tissue handling, particularly in post-mortem environments (19). Recently, we and others successfully used matrix-assisted laser desorption/ionization imaging mass spectrometry (MALDI-IMS) to determine the spatial distribution of ATP within tissues while maintaining tissue and molecular integrity (20–23). However, these studies could not discriminate between extra- and intracellular ATP pools.

Lymphocyte migration within lymphoid tissues is critical for immunological surveillance and the effector functions of lymphocytes (24). A study by Pham reported an interesting antagonistic relationship between C-C chemokine receptor type 7 (CCR7) ligand chemokines and sphingosine-1-phosphate (S1P) in naïve T cell migration, whereby T cells balanced CCR7-mediated retention signals against S1P-mediated exit signals to determine their migratory behavior within LNs (25). However, what regulates these retention and/or egress promoting signals *in situ* remains incompletely resolved.

Using MALDI-IMS, the current study indicates that ATP is constitutively present in the paracortex of uninflamed LNs and that a substantial proportion of the paracortical ATP exists as eATP in the extracellular compartment. Furthermore, we provide evidence that paracortical eATP produced by chemokine-stimulated naïve lymphocytes limits CCR7-mediated cell migration *via* P2X7 receptor (P2X7R). These results collectively indicate that autocrine purinergic signaling has a homeostatic function in resting LNs.

## Materials & Methods

### Mice

C57BL/6 mice, BALB/c mice, and nude mice on the BALB/c background, and Lewis rats were purchased from Japan SLC (Izu, Shizuoka, Japan) or Charles River Laboratories (Yokohama, Japan) and housed under specific pathogen-free conditions. CCR7-deficient mice (26) were a generous gift from Dr. Martin Lipp at the Department of Tumor Genetics and Immunogenetics, Max-Delbrück-Center of Molecular Medicine, Germany. The animal experiments conducted in Osaka were performed under an experimental protocol approved by the Ethics Review Committee for Animal Experimentation of the Osaka University Graduate School of Medicine, and those conducted in Wakayama Medical University, Niigata University, and Dokkyo Medical University were approved by the Animal Research Committee of Wakayama Medical University, Niigata University, and Dokkyo Medical University, respectively, and in accordance with Japanese Government Law (No. 105). Those conducted in Turku University were approved by the Ethical Committee for Animal Experimentation (under license number 5587/04.10.07/2014) in Finland and were performed according to the 3R-principle and in adherence with the Finnish Act on Animal Experimentation (497/2013).

### Reagents and Antibodies

Polyclonal guinea pig anti-mouse CD39 and polyclonal rabbit anti-rat CD73 were obtained from J. Sevigny’s research laboratory (Université Laval, Quebec, Canada). Anti-PNAd (MECA-79) mAb was purified using a column with size-exclusion resin (Toyopearl TSK HW55; Tosoh) from the ascites of mice inoculated with the hybridoma. Purified MECA-79 was labeled with the Alexa Fluor 488 Protein Labeling Kit (Thermo Fisher Scientific). FITC anti-CD3 (145-2C11), Alexa Fluor 700 anti-CD45 (30-F-11), efluor 506 anti-MHC Class II (M5/114.15.2), eFluor 660 anti-Lyve-1 (50–0443–82), Alexa Fluor 488 anti-CD11c (N418), PE anti-CD31 (390), and Super Bright 436 anti-gp38 (8.1.1) monoclonal antibodies (mAbs) were purchased from Thermo Fisher Scientific. BV421 anti-CD8 (53-6.7), BV421 anti-CD44 (IM7), BV605 anti-CD62L (MEL-14), Alexa Fluor 647 anti-CD4 (GK1.5), PE anti-CD4 (GK1.5), FITC anti-CD8 (53-6.7), PE anti-CD49d (R1-2), PE anti-B220 (RA3-6B2), PE/Cy7 anti-CD39 (Duha59), Alexa Fluor 647 anti-CD73 (TY/11.8), biotin anti-B220 (RA3-6B2) mAbs and streptavidin-Dylight 649 were purchased from BioLegend, and biotin anti-P2X7 mAb, 1F11 (27) was a generous gift from Dr. Yosuke Kurashima (Chiba University Graduate School of Medicine). Purified anti-L-selectin mAb (MEL-14) was purchased from Bio X Cell. Streptavidin-Alexa Fluor 405 and Alexa Fluor 647 anti-rabbit IgG pAb were purchased from Thermo Fisher Scientific. Apyrase (A6535) and ATP (A7699, A2283) were purchased from Sigma-Aldrich. A small molecular fluorescent ATP probe, 2-2Zn (II), was generated as described in our previous report (28). A-438079 was purchased from Wako, and NF023 and 5-BDBD were from Tocris Bioscience.

### Immunohistochemistry

For the staining of CD39 and CD73, frozen tissue sections were fixed in methanol, and blocked with PBS containing 3% bovine serum albumin (BSA), 20 μg/ml mouse Ig, and 20 μg/ml goat IgG. The sections were incubated with anti-CD39 polyclonal antibodies (pAb) and anti-CD73 pAb, followed by Cy3 F(ab’)_2_ anti-guinea pig IgG pAb (Jackson ImmunoResearch) and Alexa Fluor 594 anti-rabbit IgG pAb (Thermo Fisher Scientific), respectively. After being blocked with rat serum, the sections were further incubated with Alexa Fluor 488 anti-PNAd mAb and biotin anti-B220 mAb, followed by Streptavidin-Alexa Fluor 405. Histochemical staining was analyzed using an FV1000-D confocal laser-scanning microscope (Olympus).

### Enzyme Histochemistry

The localization of ATPase, ADPase, or AMPase activity was determined in non-fixed murine LN cryosections using a modification of the lead nitrate Pb(NO_3_)_2_ method, as described previously (18). Tissue sections were also stained with hematoxylin and eosin. Slides were mounted with Eukitt mounting medium (Biolab), and images were acquired using an Olympus BX60 microscope.

### Exsanguination

Mice were anesthetized and exsanguinated by a right atrial cut, and LNs were subsequently harvested immediately. As negative control for this procedure, mice were sham-operated, and LNs were harvested, allowing identical time to pass before LN harvest.

### Transcardial Perfusion

Mice were anesthetized, exsanguinated by a right atrial cut as described above, transcardially perfused through a ventricular catheter with 10 ml PBS, and then the perfusion solution was allowed to drain through the incision in the atrium. As negative control for this procedure, mice were sham-operated, and LNs were harvested, allowing identical time to pass before LN harvest.

### Afferent Lymphatic Vessel Interruption

Mice were anesthetized with isoflurane and subjected to occlusion of the afferent lymphatics of popliteal LNs, as described previously (29). Briefly, popliteal LNs were exteriorized, and afferent lymphatic vessels were electro-cauterized, leaving efferent lymphatic vessels and blood vessels intact. Mice were sacrificed at different timepoints after afferent lymphatic vessel interruption. The contralateral popliteal LNs were used as unoperated control tissues.

### Quantitation of Adenine Nucleotides in the Thoracic Duct Lymph

Thoracic duct lymph from Lewis rats was obtained by routine thoracic duct cannulation and collected aseptically in EDTA-containing tubes every 15 min at 4°C. The quantitation of adenine nucleotides was performed by ion chromatography-electrospray ionization-mass spectrometry (IC-ESI-MS) as described below.

### MALDI-IMS

The visualization of adenine nucleotides by imaging mass spectrometry was performed as described previously (22). To minimize the hydrolysis of adenine nucleotides because of postmortem ischemia, LNs were rapidly collected under anesthesia, embedded in SCEM (Leica Microsystems), and snap frozen in liquid nitrogen. Frozen tissue blocks sectioned at a thickness of 8 μm using a cryostat (CM3050, Leica Microsystems) were thaw-mounted onto indium tin oxide coated glass slides (Bruker Daltonics, MA, USA). Matrix (10 mg/ml 9-aminoacridine dissolved in 70% ethanol) was manually coated onto the slides using an artistic airbrush (Procon Boy FWA Platinum 0.2-mm caliber airbrush, Mr. Hobby, Tokyo, Japan) as described previously (30). MALDI-IMS analysis was performed with a TOF/TOF-mass spectrometer (Ultraflextreme, Bruker Daltonics, Bremen, Germany) equipped with a 355-nm Nd:YAG laser. Ion signals for ATP, ADP, and AMP observed at *m/z* 506.0, 426.0, and 346.1 respectively, were monitored at every spectrum obtained with a raster scan pitch of 50 μm, on the tissue surface. Thereafter, the spectral data were transformed to image data and analyzed using Flex imaging and SCiLS 2019a (Bruker Daltonics) software. Serial sections were used for immunohistochemical analysis by staining with fluorochrome-conjugated monoclonal antibodies, and examined by confocal microscopy (LSM710; Zeiss).

### IC-ESI-MS

Adenine nucleotides in tissues and fluids were quantified using an orbitrap-type MS (Q Exactive Focus, Thermo Fisher Scientific), connected to a high performance ion-chromatography system (ICS-5000+, Thermo Fisher Scientific) that enabled us to perform highly selective and sensitive quantification of hydrophilic metabolites according to the IC-separation and Fourier Transfer MS principle (31), as described previously (32). Briefly, frozen mouse LNs were homogenized in methanol using a manual homogenizer (Finger Masher, AM79330, Sarstedt), followed by the addition of an equal volume of chloroform and a 0.4 volume of Milli-Q water. After centrifugation (three cycles at 4,000 rpm for 60 s), the aqueous phase was ultrafiltered using an ultrafiltration tube (Ultrafree-MC, UFC3 LCC NB, Human Metabolome Technologies), and the filtrates were dried. The dried residues were redissolved in Milli-Q water and used for IC-ESI-MS. The IC was equipped with an anion electrolytic suppressor (Thermo Scientific Dionex AERS 500) to convert the potassium hydroxide gradient into pure water before the sample entered the mass spectrometer. The separation was performed using a Thermo Scientific Dionex IonPac AS11-HC, 4 μm particle size column. The IC flow rate was 0.25 mL/min supplemented post-column with 0.18 mL/min makeup flow of MeOH. The potassium hydroxide gradient conditions for IC separation were as follows: from 1 mM to 100 mM (0–40 min), 100 mM (40–50 min), and 1 mM (50.1–60 min), at a column temperature of 30°C. The Q Exactive Focus mass spectrometer was operated under an ESI negative mode for all detections. Full mass scan (*m/z* 70−900) was used at a resolution of 70,000. The automatic gain control target was set at 3 × 10^6^ ions and the maximum ion injection time was 100 ms. Source ionization parameters were optimized at 3 kV spray voltage and other parameters as follows: transfer temperature was 320°C, S-Lens level was 50, heater temperature was 300°C, sheath gas was 36, and Aux gas was 10.

### Apparent Contents of ATP in LNs

Serial sections (8 μm; ×20) of tissues subjected to imaging MS were collected in tubes, from which metabolites were extracted using the method described above. For these tissue sections, the absolute concentration of adenine nucleotides per tissue weight was quantified by IC-ESI-MS. Using the ATP concentrations in the proximal tissues and the tissue fractionation maps of ATP levels obtained by imaging MS, we created apparent content maps of ATP by applying the equations described previously (21).

### Chemotaxis Assay

This was performed as described previously (33) with minor modifications. Briefly, cells were isolated from inguinal LNs and mesenteric LNs, and then starved in RPMI-1640 medium containing 0.1% BSA for 30 min (starvation usually make cells respond better to chemokines). Serum-starved LN cells were added to the upper chamber of a 24-well plate containing Transwell inserts with a pore size of 5.0 μm (Corning) together with various doses of ATP. 100 ng/ml CCL19 (R&D Systems), 100 ng/ml CCL21 (BioLegend), or 10 nM S1P (Sigma-Aldrich) were applied to the lower chamber in RPMI-1640 medium containing 0.1% BSA. After a 2 h incubation at 37°C in a CO_2_ incubator, the cells in the lower chamber were stained with Alexa Fluor 647 anti-CD4, BV421 anti-CD8, and PE anti-CD49d, and then the number of cells that had migrated into the lower chamber was counted by FACSverse (BD Biosciences). In the P2X antagonist experiments, serum-starved LN cells were treated with 10 μM of A-438079 (Wako Pure Chemical), NF023 (Tocris Bioscience), or 5-BDBD (Tocris Bioscience) for 30 min at 37°C (12) before adding these cells to the upper chamber. In the apyrase treatment experiments, apyrase was first dialyzed with Amicon Ultra 30K (Merck) against saline, and 2.5 units apyrase were then added to 500 nmol ATP and left for 30 min at 30°C. The apyrase-treated ATP was subsequently added to the lower chamber.

### Live Cell Imaging of ATP Release

Real-time imaging of ATP release on the cell surface was performed as previously described (28) with some modifications. Briefly, cells suspended in HEPES-buffered saline were stained for 5 min with 1 μM of 2-2Zn(II) and then stimulated with CCL19 (100 ng/ml) or CXCL12 (100 ng/ml). Fluorescence live-cell imaging was performed with an inverted Olympus IX71 microscope (Olympus) and a Roper Scientific RS Photometrics Cool Snap CF camera (Roper Scientific). Fluorescence images were captured with ×100 oil objectives (NA 1.4) using YFP filter sets and MetaVue research imaging software (Molecular Devices). Image analysis was performed with ImageJ software.

### *In Vivo* P2X7R Inhibition by A-438079

Osmotic minipumps (model 2001D; Alzet, Durect) were implanted into mice subcutaneously, and the P2X7R antagonist A-438079 (32 mg/ml) or vehicle alone (sterile saline with 27% DMSO) was administered through the pump at a rate of 7.85 μl/h for 22 h. The dose of A-438079 used was based on previous studies (34, 35). Preliminary studies indicated that this treatment did not inhibit the migration of CD4^+^ or CD8^+^ T cells into LNs (**Supplementary Figure 1**). Subsequently, T cell retention was assessed in LNs as described previously with minor modifications (25). Briefly, 2 h later, 1 × 10^7^ CFSE-labeled lymphocytes (mixture of splenic and mesenteric LN cells) were injected i.v. into recipients, and after 2 h, anti-L-selectin antibody (MEL-14, 200 μg) was administered i.p to block the entry of additional new lymphocytes. Twenty hours post LN entry blockade, the inguinal LNs were harvested, and LN cells were stained with PE anti-CD49d, Alexa Flour 647 anti-CD4, and BV421 anti-CD8 antibodies for analysis by flow cytometry. Subsequently, the number of CFSE-labeled CD4^+^ or CD8^+^ naïve T cells was determined using Count Bright Absolute Counting Beads (Thermo Fischer Scientific) after gating on the CD49d^lo^ cell fraction.

### Measurement of CD39, CD73 and P2X7R Expression Levels by FACS

LNs cells were collected from the inguinal lymph nodes, blocked with TruStain FcX (Biolegend), and stained with FITC anti-CD3, BV421 anti-CD8, BV421 anti-CD44, BV605 anti-CD62L, Alexa Fluor 647 anti-CD4, PE anti-CD4, FITC anti-CD8, PE anti-CD49d, PE anti-B220, PE/Cy7 anti-CD39, Alexa Fluor 647 anti-CD73 antibodies, and biotin anti-P2X7 antibody followed by streptavidin-Dylight649. T and B cells were identified for their expression of CD3 and B220, respectively. For dendritic cells and stromal cells, we used the method we reported previously (36) with some modifications. Briefly, LNs were isolated from mice, and a single-cell suspension was prepared with collagenase D (Roche) and DNase I (Roche) in RPMI-1640 medium (Wako), blocked with TruStain FcX, and stained with Alexa Fluor 700 anti-CD45, efluor 506 anti-MHC Class II, Alexa Fluor 488 anti-CD11c, PE anti-CD31, Super Bright 436 anti-gp38 antibodies, and biotin anti-P2X7 antibody followed by streptavidin-Dylight649. Data acquisition was carried out using FACSVerse or FACSCelesta flow cytometer (BD Biosciences); the data were analyzed with FlowJo software (Tree Star Inc.).

### Effect of ATP on T Cell Viability

LN cells were incubated in the presence or absence of ATP in 0.1% BSA containing RPMI1640 for 2 h at 37°C. We prepared dead cells by heat treatment as described in our previous report (37) with some modifications. Briefly, LN cells were incubated at 56°C for 10 min, and then at 37°C for 2 h to induce cell death. The LN cells were stained with BV421 anti-CD8, Alexa Flouor647 anti-CD4, and PE anti-CD49d. The extent of cell death was examined using 7-AAD (Biolegend), and the data were acquired using the FACSCelesta flow cytometer.

### Statistical Analysis

Differences between groups were evaluated by Student’s *t*-test for single comparisons or one-way ANOVA, followed by *post-hoc* Tukey tests, for multiple comparisons. The statistical analyses were performed using Prism software (GraphPad). A *p*-value < 0.05 was considered statistically significant. Data are presented as the mean ± SD unless otherwise indicated.

## Results

### ATP and ATP-Degrading Enzymes Are Expressed in Resting LNs and a Fraction of the ATP Resides in the Extracellular Compartment

**Figure 1:**
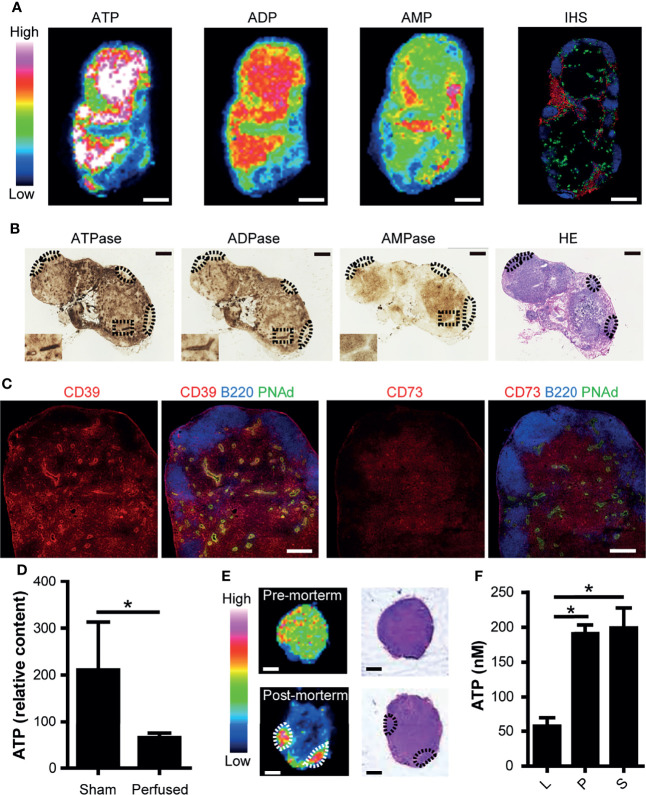
ATP and ATP-degrading enzymes are constitutively expressed in the paracortex of uninflamed LNs. **(A)** Imaging mass spectrometry analysis of adenine nucleotides in uninflamed LN. Serial sections of inguinal LNs were used for MALDI-IMS imaging and immunohistochemical (IHC) analyses. Representative MALDI-IMS images for ATP, ADP, and AMP in LN from three individual experiments are shown. Rainbow color scales were used. The white color represents the highest signal intensity (100%) and the black color represents the lowest signal (0%) of a specific ion. A serially-cut cryosection of LN was stained for B220 (blue), PNAd (green), and Lyve-1 (red). Scale bar, 500 μm. **(B)** Enzyme histochemical staining for ATPase, ADPase, and AMPase. The inset shows a representative area in the paracortex with an HEV in the middle. B cell follicles are indicated by dotted circles. Scale bar, 500 μm. **(C)** Confocal microscopic images of an inguinal LN stained for CD39 and CD73. On the left, a cryosection was stained for CD39 (red), B220 (blue), and PNAd (green). On the right, a serially-cut section was stained for CD73 (red), B220 (blue), and PNAd (green). Scale bar, 200 μm. **(D)** ATP content in inguinal LNs before and after transcardial perfusion with PBS, as determined by capillary electrophoresis-electrospray ionization-mass spectrometry. Data represent the mean ± SEM from at least three independent experiments. *p<0.05 by Student’s *t*-test. **(E)** IMS analysis of ATP (left column) and H&E staining (right column) of inguinal LNs *pre-mortem* and *post-mortem*. **(F)** ATP content in thoracic duct lymph (L), plasma (P), and serum (S). Data represent the mean ± SEM from three independent experiments. *p<0.05 by one-way analysis of variance followed by Tukey’s multiple comparison test, compared with L.

To examine whether purine nucleotides including ATP are constitutively present in resting LNs, we performed MALDI-IMS analysis (22) and obtained molecular images of ATP (*m/z* 506), ADP (*m/z* 426), and AMP (*m/z* 346) on frozen sections of inguinal LNs from specific-pathogen-free mice. As shown in **Figure 1A**, ATP and ADP were mainly detected in the T cell area of the paracortex and less prominently in the B cell follicles of uninflamed LNs. In contrast, AMP was present in areas where ATP and ADP were relatively scarce.

Consistent with these findings, enzyme histochemical analysis showed that ATPase and ADPase were abundant in the T cell area of resting LNs, whereas AMPase was less abundant but still localized in T cell areas (**Figure 1B**). An area close to the high endothelial venules (HEVs) showed particularly strong ATPase and ADPase activities, consistent with our previous report (18). Immunohistochemical analysis demonstrated that CD39 (ecto-ATP/ADPase) and CD73 (ecto-AMPase) were strongly expressed in the T cell area, confirming the above results of enzymatic histochemistry (**Figure 1C**). To determine which cell types express CD39 and CD73, we performed flow cytometric analysis. While CD39 and CD73 were expressed in all cell types we analyzed, CD39 was strongly expressed on the surface of dendritic cells (DCs), whereas CD73 was strongly expressed on T cells (**Supplementary Figure 2**). These observations are compatible with the idea that eATP is constitutively produced in the T cell area of uninflamed LNs but is degraded mainly *in situ* by ecto-nucleotidases constitutively expressed on dendritic cells and T cells in the same area.

To examine whether the ATP detected above was derived from the extracellular compartment, we examined ATP production in LNs before and after the transcardial perfusion of mice with phosphate buffered saline. As shown in **Figure 1D**, transcardial perfusion strongly reduced ATP levels in LNs, in agreement with the idea that a substantial proportion of ATP exists freely in the extracellular compartment of LNs. Supporting this, exsanguination also significantly reduced the ATP signals in LNs (**Supplementary Figure 3**). Furthermore, at 10 min post-mortem, ATP signals in the T cell area decreased rapidly, whereas ATP signals in the B cell follicles were relatively unchanged (**Figure 1E**). These results are compatible with the idea that ATP in the T cell area is more labile than ATP in the B cell area, in agreement with the hypothesis that a substantial proportion of ATP exists as eATP in the T cell area. This observation is also in accord with stronger ATPase activity in the T cell area compared with the B cell area (**Figures 1B, C**).

Then, we examined whether eATP leaves LNs *via* the efferent lymph. To this end, we used rats because thoracic duct lymph collection is relatively easy because of their large size. ATP was present in the thoracic duct lymph, although at several timepoints, lower levels were measured compared with the serum and plasma (**Figure 1F**). This is in agreement with the idea that a proportion of eATP leaves LNs in a non-degraded form *via* the efferent lymph, entering the thoracic duct to return ultimately to the systemic circulation.

### T Cells Are Required for Paracortical ATP Signals

**Figure 2:**
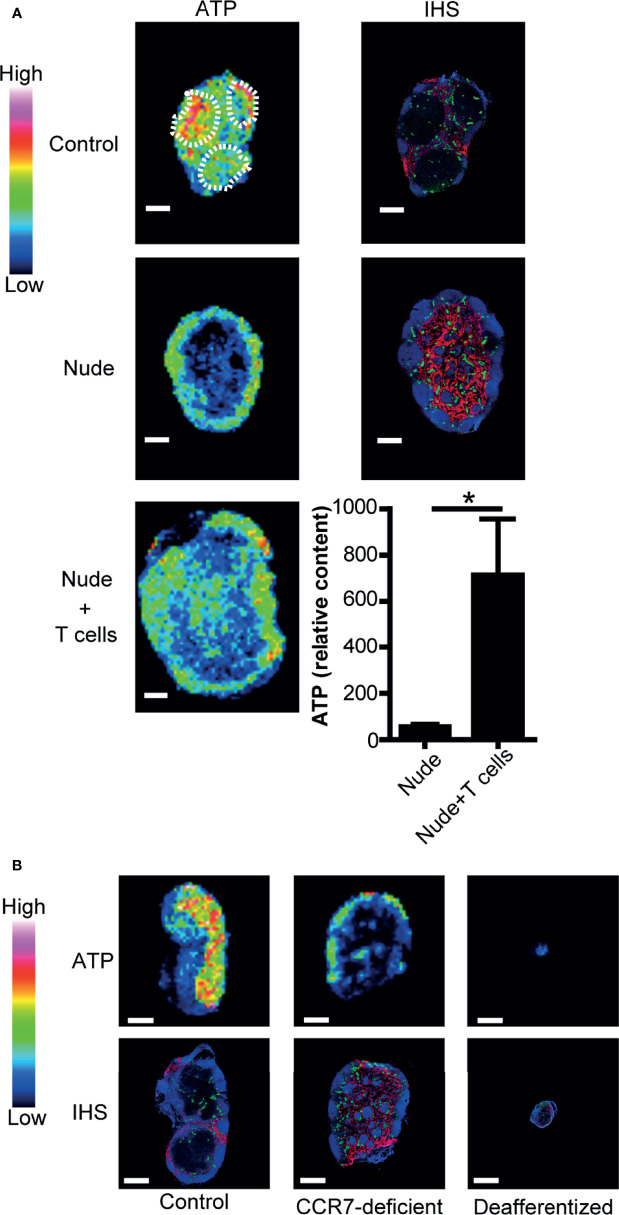
Naïve T cells are required for ATP production in LNs. **(A)** IMS analysis of ATP and immunohistochemistry of LNs from control mice (upper column) and nude mice (middle column). B220 (blue), PNAd (green), and Lyve-1 (red) were stained by immunohistochemistry (right column). The lower column shows the quantitative imaging of ATP (left) and ATP content (right) in the inguinal LNs of nude mice 2 days after adoptive T cell transfer. Scale bar, 500 μm. T cell areas are indicated by dotted circles. Bar graphs indicate relative content of ATP signals. Data represent the mean ± SD of four (nude + T cells) or five (nude) experiments. *p<0.05 by Student’s *t*-test. **(B)** IMS analysis of ATP (upper column) and immunohistochemistry (lower column) in the inguinal LNs of control mice (left), CCR7-deficient mice (center), and mice subjected to the ligation of afferent lymphatics of the inguinal LNs (right). Scale bar, 500 μm. The result shown is representative of three independent experiments.

We next examined how ATP expression is regulated in the LN paracortex. As shown in **Figure 2A**, nude mice had low ATP signals in the paracortex where naïve T cells are deficient (38) but substantial ATP signals in the B cell follicles. Upon adoptive T cell transfer, ATP content in the LN was markedly increased, and there was a substantial increase in ATP signals in the T cell repopulated paracortex in recipient nude mice (**Figure 2A**). This indicated that immigrating T cells are important for paracortical ATP expression. In addition, CCR7-deficient mice devoid of naïve T cells in the paracortex (26) showed strongly attenuated paracortical ATP expression (**Figure 2B**). We then abrogated naïve T cell accumulation in the paracortex by ligating the afferent lymphatics of popliteal LNs as reported previously (29, 39) and examined ATP signals in these LNs 4 weeks later. As expected, T cells were strongly depleted in these LNs, which were atrophic, and there was a marked reduction in the paracortical ATP signal (**Figure 2B**). These results further strengthen the idea that CCR7-expressing naïve T cells are required for the generation of ATP signals in the LN paracortex.

### LN Lymphocytes Release ATP Upon Chemokine Stimulation

**Figure 3:**
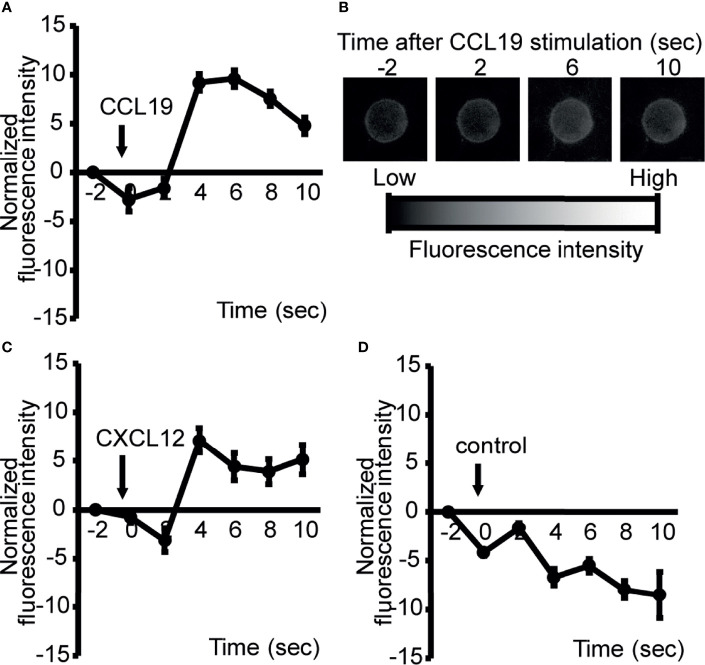
ATP is rapidly released from LN cells upon exposure to chemokines constitutively expressed in the paracortex. To image ATP release at the cell surface, LN cells were stained with a cell surface-targeting fluorescent ATP probe, 2-2Zn(II), and the response to 100 ng/ml CCL19 **(A)**, CXCL12 **(C)**, or medium alone **(D)** was analyzed by fluorescence microscopy. Representative images of CCL19-induced ATP release from LN cells **(A)**. Fluorescence changes were recorded at 2 s intervals and normalized by subtracting the baseline values at −2 s (CXCL12, n=8; CCL19, n=9). Data are representative of two independent experiments. Apparent reductions in fluorescent intensity observed in **(D)** were because of fluorescence quenching with time. Representative images of ATP release were visualized by fluorescence microscopy and analyzed over time **(B)**.

The preferential localization of ATP in the paracortex of uninflamed LNs suggests that the cells residing in this compartment produce eATP in the absence of antigenic stimulation. Previously, Ledderose et al. (12) reported that isolated human naïve CD4 T cells generated ATP upon CXCL12 chemokine stimulation *in vitro*. Given that CCR7 ligands (CCL19 and CCL21) (26) and CXCL12 (40) are constitutively expressed in the LN paracortex and that a lack of naïve T cells strongly reduced eATP expression (**Figures 2A, B**), we thought it likely that constitutively expressed chemokines including the CCR7 ligand chemokines are involved in the generation of ATP in this anatomical compartment. To investigate this further, we labeled LN lymphocytes with 2-2Zn(II), a fluorescent nucleotide-sensing probe, which detects the extracellular release of ATP *via* membrane surface (28, 41), and examined the ATP release before and after stimulation with CCR7 or CXCR4 ligand chemokines, CCL19 or CXCL12, respectively. CCL19 induced robust ATP release from LN lymphocytes within seconds of stimulation in the apparent absence of antigen receptor stimulation (**Figures 3A, B**), and similarly, CXCL12 also induced the potent release of ATP from LN cells as reported previously (12) (**Figure 3C**), whereas sham treatment did not induce ATP release (**Figure 3D**). Because most LN cells are naïve lymphocytes (42–44), these results agree with the idea that chemokines constitutively expressed in the paracortex, such as CXCL12 and CCL19, induce ATP production from naïve lymphocytes, at least transiently, in the LN paracortex.

### ATP Counteracts the Retention-Promoting CCR7 Signal, Sparing the Egress-Promoting S1P Signal to T Cells

**Figure 4:**
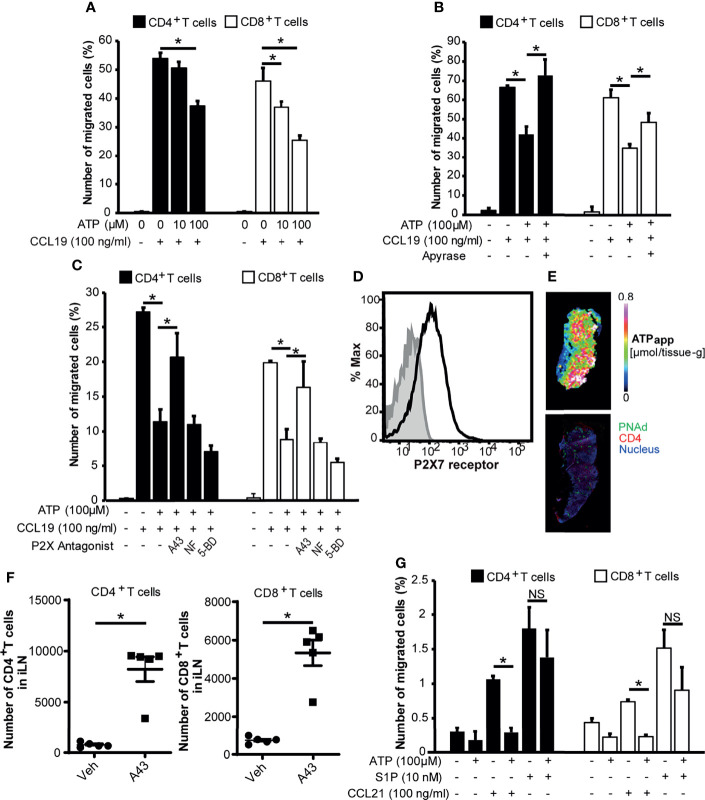
ATP inhibits CCL19-induced chemotaxis but not S1P-induced chemotaxis of CD4^+^ T cells and CD8^+^ T cells *via* P2X7R. **(A)** CCL19-dependent T cell chemotaxis in the presence or absence of ATP was analyzed by Transwell assay. The result shown is representative of two independent experiments. Data represent the mean ± SD of triplicate wells. *p<0.05 by Student’s *t*-test. **(B)** The effect of ATP on CCL19-dependent T cell chemotaxis in the presence of ATP or apyrase-treated ATP in the lower well. The result shown is representative of three independent experiments. Data represent the mean ± SD of triplicate wells. *p<0.05 by Student’s *t*-test. **(C)** T cell chemotaxis in the presence of ATP and P2 receptor antagonists. CD4^+^ and CD8^+^ T cells were treated with A-438079 (P2X7R inhibitor; 10 μM), NF023 (P2X1R inhibitor; 10 μM), or 5-BDBD (P2X4R inhibitor; 10 μM) and subjected to the CCL19-dependent chemotaxis assay in the presence or absence of 100 μM ATP in the lower well. The result shown is representative of two independent experiments. Data represent the mean ± SD of triplicate wells. *p<0.05 by Student’s *t*-test. **(D)** Expression of P2X7R on CD3^+^ CD49d^low^ T cells in LNs analyzed by flow cytometry. P2X7R is shown in black. Isotype control is shown in grey. The result shown is representative of at least three independent experiments. **(E)** An apparent contents map of ATP (ATP_app_) generated as previously described (21) and a confocal microscopic image of a serial LN section stained for CD4 (red), PNAd (green), and nucleus (blue). Images are representative of three individual experiments. **(F)** The effect of the P2X7R antagonist A-438079 on the intranodal retention of circulating lymphocytes. Data represent the mean ± SEM of five mice. *p<0.05 by Student’s *t*-test. **(G)** The effect of ATP on S1P-dependent T cell chemotaxis. S1P or CCL21 was added to the lower wells in the presence or absence of ATP at the indicated concentrations, and T cells were added to the upper wells. Relative cell migration was determined as described above. The result shown is representative of two independent experiments. Data represent the mean ± SD of triplicate wells. *p<0.05 by Student’s *t*-test; NS, not significant.

We next examined the biological role of eATP in the LN paracortex. Because preliminary analysis indicated that ATP induced very little chemotaxis in naïve lymphocytes, we investigated whether ATP has regulatory roles in lymphocyte migration rather than providing chemotactic cues in LNs. To this end, we examined the effect of ATP on the chemokine-induced migration of CD4^+^ T cells and CD8^+^ T cells. As shown in **Figure 4A**, ATP inhibited the CCL19-induced chemotaxis of CD4^+^ and CD8^+^ T cells at high micromolar concentrations in a dose-dependent manner, which was abrogated by apyrase treatment (**Figure 4B**), whereas preliminary experiments showed that apyrase alone did not affect CCL19-induced chemotaxis. To exclude the possibility that ATP inhibited T cell chemotaxis by inducing cell death, we examined the T cell viability upon addition of ATP. We found that 100 μM ATP that inhibited CCL19-induced chemotaxis did not affect T cell viability (**Supplementary Figure 4**). These results indicated that ATP itself inhibits CCL19-dependent T cell chemotaxis without affecting T cell viability. The inhibitory effects were significantly attenuated by a P2X7R-specific antagonist A-438079 (A43) but not by a P2X1R antagonist NF023 (NF) or a P2X4R antagonist, 5-BDBD (5-BD) (**Figure 4C**). This suggests that ATP limits the CCR7-mediated chemotaxis of naïve T cells *via* P2X7R. We also found that naïve LN T cells expressed P2X7R on their cell surface (**Figure 4D** and **Supplementary Figure 5**), although P2X7R was more highly expressed on memory T cells compared with naïve LN T cells, supporting several previous reports (45–47).

Because P2X7R is distinct from other P2X receptors in that it has a particularly high threshold for activation (i.e., it requires high micromolar ATP concentrations) (3), we investigated whether such concentrations of ATP exist in the LN parenchyma. To this end, we performed MALDI-IMS combined with quantitative ion chromatography electrophoresis-electrospray ionization mass spectrometry to create an apparent content map of ATP showing (μmol/tissue-g) for each pixel (20–22). We found that the apparent ATP content in the T cell area was in the order of 0.6 to 0.7 μmol/g tissue in LN sections (**Figure 4E**). Based on the reported lymph node density (1040 g/L) (48), the ATP concentration was calculated to be in the order of 600 to 700 μM, a sufficiently high concentration to activate P2X7R (EC_50_ > 100 μM) (34).

Because these results suggest that ATP counteracts CCR7-mediated chemotactic signals *via* P2X7R, we examined the role of P2X7R role *in vivo*. As shown in **Figure 4F**, the administration of a P2X7R antagonist A-438079 enhanced the intranodal retention of circulating lymphocytes, suggesting that the blockade of eATP signals promoted CCR7-mediated T cell retention in LNs. After the treatment the mice looked healthy, and no apparent macroscopic changes or adverse effects were observed. We then examined whether ATP affected egress-promoting signals provided by S1P. As shown in **Figure 4G** and **Supplementary Figure 6**, ATP did not affect S1P-dependent T cell chemotaxis significantly, although it affected T cell migration induced by another CCR7 ligand, CCL21, in agreement with the idea that ATP preferentially limits CCR7 signals but not egress-promoting S1P signals.

## Discussion

Although T cell receptor ligation induces ATP release from T cells in lymphoid tissues (8–10), studies by Lazarowski et al. (14, 49, 50) showed that the non-lytic release of ATP occurred in most tissues and cell types under physiological conditions. The present study indicate that the non-lytic release of ATP also occurs in lymphoid tissues in the steady state. Using MALDI-IMS analysis, we found constitutive ATP signals mainly in the paracortical T cell area of uninflamed LNs, which was strongly diminished by diluting the extracellular fluid compartment by the transcardial perfusion of mice with phosphate buffered saline. Together with the observations that T cell depletion and the abrogation of CCR7 signaling markedly reduced paracortical ATP signals, it is likely that at least part of the ATP signal is derived from ATP released from CCR7-expressing naïve T cells. In direct support of this idea, the CCR7 ligand chemokine CCL19, which is constitutively expressed in the stroma of the T cell area, triggered a transient but robust release of ATP from LN lymphocytes *in vitro* in the apparent absence of T cell receptor ligation. Furthermore, CXCL12 expressed by T zone stromal cells also induced the rapid and robust release of ATP from LN cells *in vitro* as shown previously with human lymphocytes (12). Given that ATP was also present in the thoracic duct lymph, a proportion of the naïve T cell-derived eATP is likely to leave LNs in a non-degraded form *via* the efferent lymph, entering the thoracic duct to return ultimately to the systemic circulation.

Because the steady-state expression in the T cell area of uninflamed LNs implies a homeostatic role of eATP in this region, we examined the role of ATP in the chemokine-induced migration of CD4^+^ T cells and CD8^+^ T cells. We found that ATP inhibited the CCL19-induced chemotaxis of CD4^+^ T cells and CD8^+^ T cells at high micromolar concentrations in a dose-dependent manner, which was abrogated by the ATP-degrading enzyme apyrase. These inhibitory effects were significantly attenuated by antagonizing P2X7R, but not P2X1R or P2X4R, suggesting that ATP counteracts the CCR7-mediated chemotaxis of T cells *via* P2X7R. Supporting this idea, most naïve T cells in LNs expressed P2X7R, and a P2X7R antagonist enhanced the intranodal retention of circulating lymphocytes, which was previously shown to be partly CCR7-dependent (25). These results suggest that naïve T cell-derived ATP acts on naïve T cells in an autocrine manner *via* P2X7R, to regulate T cell migration within LNs. Currently, how P2X7 signaling affects CCR7-dependent chemotaxis remains unclear. MALDI-IMS combined with capillary electrophoresis-electrospray ionization mass spectrometry, which allows the determination of the apparent contents of adenine nucleotides in serial tissue sections (21), confirmed the presence of high micromolar concentrations of ATP in the T cell area of uninflamed LNs. Although numerous studies have demonstrated that ATP is a proinflammatory molecule that enhances immune responses upon ligation with P2X7R expressed by immune cells (51), our study indicates that ATP is also involved in the homeostatic regulation of T cell migration *in vivo via* interactions with P2X7R.

The current dogma concerning lymphocyte migration within LNs dictates that CCR7 ligand chemokines provide retention signals to T cells, whereas S1P promotes T cell egress from LNs by overcoming the CCR7-mediated signals (24). Our study suggests that eATP might add another level of complexity to this scenario, whereby homeostatic T cell movement within tissues is orchestrated by the concerted action of CCR7 ligand chemokines, S1P produced by stromal cells, and eATP produced by chemokine-stimulated naïve T cells. The involvement of ATP receptors other than P2X7R should be examined in the future. Also, while our data suggest that S1P-dependent cell migration is more resistant to inhibition by P2X7R signaling compared to CCR7 ligand-dependent cell migration, the mechanisms remain unknown. Furthermore, we found that P2X7R is also expressed on the T zone stromal cells including fibroblast reticular cells (FRCs) (**Supplementary Figure 7**). Given that a previous report has shown that FRCs regulate naïve T cell retention in the LNs (52), P2X7R on the FRC surface may also be involved in the T cell retention. These points also should be clarified in future study.

Although not addressed in the present study, eATP in the paracortex might also act on non-T cells in a paracrine manner because ATP affected the maturation (53) and migration of dendritic cells (54, 55), barrier formation by vascular endothelial cells (56) and lymphatic endothelial cells (57), macromolecular uptake (58), and bacterial killing by macrophages (59).

A limitation of the present study was that the MALDI-IMS method used might not detect extracellular ATP in the tissue stroma alone. However, if the method used also detected ATP in the intracellular compartment, it would have detected uniformly strong signals throughout the LN because all cells are thought to contain comparably high (i.e., several millimolar) concentrations of ATP (49). The selective loss of ATP signals from the T cell area but the retention of such signals in the B cell area subsequent to the transcardial perfusion or exsanguination of mice supports the idea that the ATP signals detected by the currently used method were mainly derived from the extracellular compartment and that eATP is readily metabolized or removed from the T cell area but not from the B cell area. However, it has to be kept in mind that the transcardial perfusion as well as the exsanguination might have affected ATP metabolism *in situ*.

Collectively, we provide suggestive evidence that autocrine purinergic signaling induced by the regulated release of ATP from naïve T cells in response to lymphoid chemokines has a homeostatic function in naïve T cell migration in resting LNs.

## Data Availability Statement

The original contributions presented in the study are included in the article/**Supplementary Material**. Further inquiries can be directed to the corresponding author.

## Ethics Statement

The animal study was reviewed and approved by the Ethics Committees of Osaka University, Wakayama Medical University, Niigata University, and Dokkyo Medical University.

## Author Contributions

DK, YS, HH, AT, and HU designed the study, acquired, analyzed and interpreted the data, and drafted the article. EU, SM, TK, MSu, IH, GY, MSa, and SJ analyzed and interpreted the data. MM conceived and designed the study, analyzed and interpreted the data, and drafted the article.

## Funding

This work was supported by Grants-in-Aid 24111005 (MM) and 19K16697 (DK) from the Ministry of Education, Culture, Sports, Science, and Technology of Japan, a grant from the 2018 Wakayama Medical Award for Young Researchers, and by the FiDiPro program of the Academy of Finland.

## Conflict of Interest

The authors declare that the research was conducted in the absence of any commercial or financial relationships that could be construed as a potential conflict of interest.

## Publisher’s Note

All claims expressed in this article are solely those of the authors and do not necessarily represent those of their affiliated organizations, or those of the publisher, the editors and the reviewers. Any product that may be evaluated in this article, or claim that may be made by its manufacturer, is not guaranteed or endorsed by the publisher.

## Supplementary Material

Supplementary Figure 1 P2X7R blockade does not affect T cell entry into LNs. 1 × 10^7^ CFSE-labeled lymphocytes were injected i.v. into recipients, and then A-438079 (200 μg/mouse) was administered i.p. Four hours later, the CFSE-labeled CD4^+^ or CD8^+^ naïve T cells in inguinal LNs were counted after gating on the CD49d^lo^ cell fraction. Data represent the mean ± SEM of four mice. NS, not significant.

Supplementary Figure 2 Expression levels of CD39 and CD73 on T cells, B cells, and DCs. Expression of CD39 **(A)** and CD73 **(B)** on CD3^+^ T cells, B220^+^ B cells, CD11c^+^ MHC class II^hi^ migratory DCs, and CD11c^+^ MHC class II^lo^ resident DCs in LNs was analyzed by flow cytometry. CD39 and CD73 are shown in red. Isotype control is shown in grey. The histograms are representative of three independent experiments. Expression levels of CD39 **(C)** and CD73 **(D)** on B cells, T cells, migratory DCs, and resident DCs are comparatively shown. The RMFI indicates the relative median fluorescence intensities. Data represent the mean ± SD of three independent experiments.

Supplementary Figure 3 Exsanguination reduces ATP levels in LNs. Mice were exsanguinated by puncture of the retro-orbital plexus under isoflurane anesthesia, and then inguinal LNs were collected. ATP contents in the LNs were determined by MALDI-IMS analysis. Data represent the mean ± SD of five or six replicates. **p*<0.05 by Student’s *t*-test.

Supplementary Figure 4 The effect of ATP on T cell viability. LN cells were treated with or without 100 μM ATP. Two hours later, the frequency of 7-AAD^+^ CD4^+^ or CD8^+^ dead T cells was evaluated after gating on CD49d^lo^ cell fraction. Heat-treated cells were used as a positive control. The results shown are representative of two independent experiments. Data represent the mean ± SD of triplicate wells. NS, not significant.

Supplementary Figure 5 P2X7 receptor expression on T cell substes in LNs. Expression of P2X7 receptor on CD62L^hi^ CD44^lo^ naïve T cells (naïve) and CD62L^lo^ CD44^hi^ effector memory T cells (memory) in LNs was analyzed after gating on CD4^+^ or CD8^+^ cell fraction by flow cytometry. P2X7R is shown in red. Isotype control is shown in grey. The histograms are representative of three independent experiments. Bar graphs indicate RMFI of P2X7 receptor on naïve and memory T cells. Data represent the mean ± SD of three independent experiments.

Supplementary Figure 6 ATP inhibits CXCL12, CCL19 and CCL21 dependent cell migration. T cell chemotaxis with or without ATP was analyzed by Transwell assay. **(A)** CD49d^lo^ CD4^+^ T cells, **(B)** CD49d^lo^ CD8^+^ T cells. The result shown is representative of two independent experiments. Data represent the mean ± SD of triplicate wells. **p*<0.05 by Student’s *t*-test.

Supplementary Figure 7 P2X7R expression on stromal cells in LNs. Expression of P2X7R on CD45^-^ gp38^+^ CD31^-^ FRCs, CD45^-^ gp38^-^ CD31^+^ BECs and CD45^-^ gp38^+^ CD31^+^ LECs in LNs was analyzed by flow cytometry. P2X7R is shown in red. Isotype control is shown in grey. The result shown is representative of two independent experiments.

## References

[1] Salmi M Yegutkin GG Lehvonen R Koskinen K Salminen T Jalkanen S. A Cell Surface Amine Oxidase Directly Controls Lymphocyte Migration. Immunity (2001) 14:265–76. doi: 10.1016/s1074-7613(01)00108-x 11290336

[2] Bours MJ Swennen EL Di Virgilio F Cronstein BN Dagnelie PC. Adenosine 5'-Triphosphate and Adenosine as Endogenous Signaling Molecules in Immunity and Inflammation. Pharmacol Ther (2006) 112:358–404. doi: 10.1016/j.pharmthera.2005.04.013 16784779

[3] Junger WG. Immune Cell Regulation by Autocrine Purinergic Signaling. Nat Rev Immunol (2011) 11:201–12. doi: 10.1038/nri2938 PMC4209705 21331080

[4] Linden J Koch-Nolte F Dahl G. Purine Release, Metabolism, and Signaling in the Inflammatory Response. Annu Rev Immunol (2019) 37:325–47. doi: 10.1146/annurev-immunol-051116-052406 30676821

[5] Granstein RD Ding W Huang J Holzer A Gallo RL Di Nardo A. Augmentation of Cutaneous Immune Responses by ATP Gamma S: Purinergic Agonists Define a Novel Class of Immunologic Adjuvants. J Immunol (2005) 174:7725–31. doi: 10.4049/jimmunol.174.12.7725 15944274

[6] Idzko M Hammad H van Nimwegen M Kool M Willart MA Muskens F. Extracellular ATP Triggers and Maintains Asthmatic Airway Inflammation by Activating Dendritic Cells. Nat Med (2007) 13:913–9. doi: 10.1038/nm1617 17632526

[7] Matsuo K Nishiuma S Hasegawa Y Kawabata F Kitahata K Nakayama T. Vaccination With Antigen Combined With αβ-ATP as a Vaccine Adjuvant Enhances Antigen-Specific Antibody Production *via* Dendritic Cell Activation. Biol Pharm Bull (2016) 39:1073–6. doi: 10.1248/bpb.b16-00087 27251512

[8] Schenk U Westendorf AM Radaelli E Casati A Ferro M Fumagalli M. Purinergic Control of T Cell Activation by ATP Released Through Pannexin-1 Hemichannels. Sci Signal (2008) 1:ra6. doi: 10.1126/scisignal.1160583 18827222

[9] Yip L Woehrle T Corriden R Hirsh M Chen Y Inoue Y. Autocrine Regulation of T-Cell Activation by ATP Release and P2X7 Receptors. FASEB J (2009) 23:1685–93. doi: 10.1096/fj.08-126458 PMC2718802 19211924

[10] Wang CM Ploia C Anselmi F Sarukhan A Viola A. Adenosine Triphosphate Acts as a Paracrine Signaling Molecule to Reduce the Motility of T Cells. EMBO J (2014) 33:1354–64. doi: 10.15252/embj.201386666 PMC4194124 24843045

[11] Ledderose C Bao Y Ledderose S Woehrle T Heinisch M Yip L. Mitochondrial Dysfunction, Depleted Purinergic Signaling, and Defective T Cell Vigilance and Immune Defense. J Infect Dis (2016) 213:456–64. doi: 10.1093/infdis/jiv373 PMC4704665 26150546

[12] Ledderose C Liu K Kondo Y Slubowski CJ Dertnig T Denicolo S. Purinergic P2X4 Receptors and Mitochondrial ATP Production Regulate T Cell Migration. J Clin Invest (2018) 128:3583–94. doi: 10.1172/JCI120972 PMC6063471 29894310

[13] Trautmann A. Extracellular ATP in the Immune System: More Than Just a "Danger Signal. Sci Signal (2009) 2:pe6. doi: 10.1126/scisignal.256pe6 19193605

[14] Lazarowski ER Boucher RC Harden TK. Constitutive Release of ATP and Evidence for Major Contribution of Ecto-Nucleotide Pyrophosphatase and Nucleoside Diphosphokinase to Extracellular Nucleotide Concentrations. J Biol Chem (2000) 275:31061–8. doi: 10.1074/jbc.M003255200 10913128

[15] Yegutkin GG Mikhailov A Samburski SS Jalkanen S. The Detection of Micromolar Pericellular ATP Pool on Lymphocyte Surface by Using Lymphoid Ecto-Adenylate Kinase as Intrinsic ATP Sensor. Mol Biol Cell (2006) 17:3378–85. doi: 10.1091/mbc.e05-10-0993 PMC1525232 16707571

[16] Di Virgilio F Sarti AC Falzoni S De Marchi E Adinolfi E. Extracellular ATP and P2 Purinergic Signalling in the Tumour Microenvironment. Nat Rev Cancer (2018) 18:601–18. doi: 10.1038/s41568-018-0037-0 30006588

[17] Yegutkin GG. Nucleotide- and Nucleoside-Converting Ectoenzymes: Important Modulators of Purinergic Signalling Cascade. Biochim Biophys Acta (2008) 1783:673–94. doi: 10.1016/j.bbamcr.2008.01.024 18302942

[18] Yegutkin GG Auvinen K Rantakari P Hollmen M Karikoski M Grenman R. Ecto-5'-Nucleotidase/CD73 Enhances Endothelial Barrier Function and Sprouting in Blood But Not Lymphatic Vasculature. Eur J Immunol (2015) 45:562–73. doi: 10.1002/eji.201444856 25402681

[19] Falzoni S Donvito G Di Virgilio F. Detecting Adenosine Triphosphate in the Pericellular Space. Interface Focus (2013) 3:20120101. doi: 10.1098/rsfs.2012.0101 23853707 PMC3638417

[20] Hattori K Kajimura M Hishiki T Nakanishi T Kubo A Nagahata Y. Paradoxical ATP Elevation in Ischemic Penumbra Revealed by Quantitative Imaging Mass Spectrometry. Antioxid Redox Signal (2010) 13:1157–67. doi: 10.1089/ars.2010.3290 PMC2956403 20486758

[21] Kubo A Ohmura M Wakui M Harada T Kajihara S Ogawa K. Semi-Quantitative Analyses of Metabolic Systems of Human Colon Cancer Metastatic Xenografts in Livers of Superimmunodeficient NOG Mice. Anal Bioanal Chem (2011) 400:1895–904. doi: 10.1007/s00216-011-4895-5 PMC3098365 21479793

[22] Sugiura Y Katsumata Y Sano M Honda K Kajimura M Fukuda K. Visualization of *In Vivo* Metabolic Flows Reveals Accelerated Utilization of Glucose and Lactate in Penumbra of Ischemic Heart. Sci Rep (2016) 6:32361–4. doi: 10.1038/srep32361 PMC5007669 27581923

[23] Miyamoto S Hsu CC Hamm G Darshi M Diamond-Stanic M Declèves AE. Mass Spectrometry Imaging Reveals Elevated Glomerular ATP/AMP in Diabetes/Obesity and Identifies Sphingomyelin as a Possible Mediator. EBioMedicine (2016) 7:121–34. doi: 10.1016/j.ebiom.2016.03.033 PMC4909366 27322466

[24] Cyster JG Schwab SR. Sphingosine-1-Phosphate and Lymphocyte Egress From Lymphoid Organs. Annu Rev Immunol (2012) 30:69–94. doi: 10.1146/annurev-immunol-020711-075011 22149932

[25] Pham TH Okada T Matloubian M Lo CG Cyster JG. S1P1 Receptor Signaling Overrides Retention Mediated by Gαi-Coupled Receptors to Promote T Cell Egress. Immunity (2008) 28:122–33. doi: 10.1016/j.immuni.2007.11.017 PMC2691390 18164221

[26] Förster R Schubel A Breitfeld D Kremmer E Renner-Müller I Wolf E. CCR7 Coordinates the Primary Immune Response by Establishing Functional Microenvironments in Secondary Lymphoid Organs. Cell (1999) 99:23–33. doi: 10.1016/s0092-8674(00)80059-8 10520991

[27] Kurashima Y Amiya T Nochi T Fujisawa K Haraguchi T Iba H. Extracellular ATP Mediates Mast Cell-Dependent Intestinal Inflammation Through P2X7 Purinoceptors. Nat Commun (2012) 3:1034–45. doi: 10.1038/ncomms2023 PMC3658010 22948816

[28] Kurishita Y Kohira T Ojida A Hamachi I. Organelle-Localizable Fluorescent Chemosensors for Site-Specific Multicolor Imaging of Nucleoside Polyphosphate Dynamics in Living Cells. J Am Chem Soc (2012) 134:18779–89. doi: 10.1021/ja308754g 23098271

[29] Mebius RE Streeter PR Breve J Duijvestijn AM Kraal G. The Influence of Afferent Lymphatic Vessel Interruption on Vascular Addressin Expression. J Cell Biol (1991) 115:85–95. doi: 10.1083/jcb.115.1.85 1918141 PMC2289917

[30] Takeda A Kobayashi D Aoi K Sasaki N Sugiura Y Igarashi H. Fibroblastic Reticular Cell-Derived Lysophosphatidic Acid Regulates Confined Intranodal T-Cell Motility. elife (2016) 2:5e10561. doi: 10.7554/eLife.10561 PMC4755752 26830463

[31] Hu S Wang J Ji EH Christison T Lopez L Huang Y. Targeted Metabolomic Analysis of Head and Neck Cancer Cells Using High Performance Ion Chromatography Coupled With a Q Exactive HF Mass Spectrometer. Anal Chem (2015) 87:6371–9. doi: 10.1021/acs.analchem.5b01350 25973679

[32] Miyajima M Zhang B Sugiura Y Sonomura K Guerrini MM Tsutsui Y. Metabolic Shift Induced by Systemic Activation of T Cells in PD-1-Deficient Mice Perturbs Brain Monoamines and Emotional Behavior. Nat Immunol (2017) 18:1342–52. doi: 10.1038/ni.3867 29058703

[33] Kobayashi D Endo M Ochi H Hojo H Miyasaka M Hayasaka H. Regulation of CCR7-Dependent Cell Migration Through CCR7 Homodimer Formation. Sci Rep (2017) 7:8536–49. doi: 10.1038/s41598-017-09113-4 PMC5561199 28819198

[34] Donnelly-Roberts DL Namovic MT Han P Jarvis MF. Mammalian P2X7 Receptor Pharmacology: Comparison of Recombinant Mouse, Rat and Human P2X7 Receptors. Br J Pharmacol (2009) 157:1203–14. doi: 10.1111/j.1476-5381.2009.00233.x PMC2743839 19558545

[35] Martins JP Silva RBM Coutinho-Silva R Takiya CM Battastini AMO Morrone FB. The Role of P2X7 Purinergic Receptors in Inflammatory and Nociceptive Changes Accompanying Cyclophosphamide-Induced Haemorrhagic Cystitis in Mice. Br J Pharmacol (2012) 165:183–96. doi: 10.1111/j.1476-5381.2011.01535.x PMC3252976 21675966

[36] Takeuchi A Ozawa M Kanda Y Kozai M Ohigashi I Kurosawa Y. A Distinct Subset of Fibroblastic Stromal Cells Constitutes the Cortex-Medulla Boundary Subcompartment of the Lymph Node. Front Immunol (2018) 9:2196. doi: 10.3389/fimmu.2018.02196 30333825 PMC6176096

[37] Kobayashi D Kiguchi N Saika F Kishioka S Matsuzaki S. Insufficient Efferocytosis by M2-Like Macrophages as a Possible Mechanism of Neuropathic Pain Induced by Nerve Injury. Biochem Biophys Res Commun (2020) 525:216–23. doi: 10.1016/j.bbrc.2020.02.032 32087968

[38] Hougen HP Ropke C. Small Lymphocytes in Peripheral Lymphoid Tissues of Nude Mice. Life-Span and Distribution. Clin Exp Immunol (1975) 22:528–38. PMC1538430 1225488

[39] Hendriks HR Eestermans IL. Disappearance and Reappearance of High Endothelial Venules and Immigrating Lymphocytes in Lymph Nodes Deprived of Afferent Lymphatic Vessels: A Possible Regulatory Role of Macrophages in Lymphocyte Migration. Eur J Immunol (1983) 13:663–9. doi: 10.1002/eji.1830130811 6884423

[40] Miyasaka M Tanaka T. Lymphocyte Trafficking Across High Endothelial Venules: Dogmas and Enigmas. Nat Rev Immunol (2004) 4:360–70. doi: 10.1038/nri1354 15122201

[41] Ledderose C Bao Y Zhang J Junger WG. Novel Method for Real-Time Monitoring of ATP Release Reveals Multiple Phases of Autocrine Purinergic Signalling During Immune Cell Activation. Acta Physiol (2015) 213:334–45. doi: 10.1111/apha.12435 PMC4293224 25482154

[42] Cose S Brammer C Khanna KM Masopust D Lefrançois L. Evidence That a Significant Number of Naive T Cells Enter non-Lymphoid Organs as Part of a Normal Migratory Pathway. Eur J Immunol (2006) 36:1423–33. doi: 10.1002/eji.200535539 16708400

[43] Inman CF Murray TZ Bailey M Cose S. Most B Cells in non-Lymphoid Tissues are Naïve. Immunol Cell Biol (2012) 90:235–42. doi: 10.1038/icb.2011.35 21556017

[44] Von Andrian UH M'rini C. *In Situ* Analysis of Lymphocyte Migration to Lymph Nodes. Cell Adhes Commun (1998) 6:85–96. doi: 10.3109/15419069809004463 9823458

[45] Stark R Wesselink TH Behr FM Kragten NAM Arens R Koch-Nole F. T (RM) Maintenance is Regulated by Tissue Damage *via* P2RX7. Sci Immunol (2018) 3:eaau1022. doi: 10.1126/sciimmunol.aau1022 30552101

[46] Borges da Silva H Beura LK Wang H Hanse EA Gore R Scott MC. The Purinergic Receptor P2RX7 Directs Metabolic Fitness of Long-Lived Memory CD8^+^ T Cells. Nature (2018) 559:264–8. doi: 10.1038/s41586-018-0282-0 PMC6054485 29973721

[47] Safya H Mellouk A Legrand J Le Gall SM Benbijja M Kanellopuulos-Langevin C. Variations in Cellular Responses of Mouse T Cells to Adenosine-5′-Triphosphate Stimulation Do Not Depend on P2X7 Receptor Expression Levels But on Their Activation and Differentiation Stage. Front Immunol (2018) 9:360. doi: 10.3389/fimmu.2018.00360 29535730 PMC5835135

[48] Mchintosh RL Anderson V. A Comprehensive Tissue Properties Database Provided for the Thermal Assessment of a Human at Rest. Biophys Rev Lett (2010) 05:129–51. doi: 10.1142/S1793048010001184

[49] Lazarowski ER Boucher RC Harden TK. Mechanisms of Release of Nucleotides and Integration of Their Action as P2X- and P2Y-Receptor Activating Molecules. Mol Pharmacol (2003) 64:785–95. doi: 10.1124/mol.64.4.785 14500734

[50] Lazarowski ER. Vesicular and Conductive Mechanisms of Nucleotide Release. Purinergic Signal (2012) 8:359–73. doi: 10.1007/s11302-012-9304-9 PMC3360093 22528679

[51] Rivas-Jáñez E Barrera-Avalos C Parra-Tello B Briceño P Rosemblatt MV Saavedra-Almarza J. P2X7 Receptor at the Crossroads of T Cell Fate. Int J Mol Sci (2020) 21:4937–59. doi: 10.3390/ijms21144937 PMC7404255 32668623

[52] Denton AE Roberts EW Linterman MA Fearon DT. Fibroblastic Reticular Cells of the Lymph Node are Required for Retention of Resting But Not Activated CD8+ T Cells. Proc Natl Acad Sci USA (2014) 11:12139–44. doi: 10.1073/pnas.1412910111 PMC4143042 25092322

[53] la Sala A Sebastiani S Ferrari D Di Virgilio F Idzko M Norgauer J. Dendritic Cells Exposed to Extracellular Adenosine Triphosphate Acquire the Migratory Properties of Mature Cells and Show a Reduced Capacity to Attract Type 1 T Lymphocytes. Blood (2002) 99:1715–22. doi: 10.1182/blood.v99.5.1715 11861288

[54] Idzko M Hammad H Van Nimwegen M Kool M Vos N Hoogsteden HC. Inhaled Iloprost Suppresses the Cardinal Features of Asthma *via* Inhibition of Airway Dendritic Cell Function. J Clin Invest (2007) 117:464–72. doi: 10.1172/JCI28949 PMC1783814 17273558

[55] Sáez PJ Vargas P Shoji KF Harcha PA Lennon-Duménil AM Sáez JC. ATP Promotes the Fast Migration of Dendritic Cells Through the Activity of Pannexin 1 Channels and P2X_7_ Receptors. Sci Signal (2017) 10:eaah7107. doi: 10.1126/scisignal.aah7107 29162744

[56] Jacobson JR Dudek SM Singleton PA Kolosova IA Verin AD Garcia JG. Endothelial Cell Barrier Enhancement by ATP is Mediated by the Small GTPase Rac and Cortactin. Am J Physiol Lung Cell Mol Physiol (2006) 291:L289–95. doi: 10.1152/ajplung.00343.2005 16825658

[57] Kousai A Mizuno R Ikomi F Ohhashi T. ATP Inhibits Pump Activity of Lymph Vessels *via* Adenosine A_1_ Receptor-Mediated Involvement of NO- and ATP-Sensitive K^+^ Channels. Am J Physiol Heart Circ Physiol (2004) 287:H2585–97. doi: 10.1152/ajpheart.01080.2003 15308482

[58] Schachter J Motta AP de Souza Zamorano A da Silva-Souza HA Guimarães MZP Persechini PM. ATP-Induced P2X_7_-Associated Uptake of Large Molecules Involves Distinct Mechanisms for Cations and Anions in Macrophages. J Cell Sci (2008) 121:3261–70. doi: 10.1242/jcs.029991 18782864

[59] Csóka B Németh ZH Szabó I Davies DL Varga ZV Pálóczi J. Macrophage P2X4 Receptors Augment Bacterial Killing and Protect Against Sepsis. JCI Insight (2018) 3:e99431. doi: 10.1172/jci.insight.99431 PMC5997389 29875325

