# Supplementary figures and images for: Extracellular ATP Limits Homeostatic T Cell Migration Within Lymph Nodes
Source: Front Immunol. 2021 Dec 22;12:786595. doi: 10.3389/fimmu.2021.786595 (PMC8728011; doi:10.3389/fimmu.2021.786595)

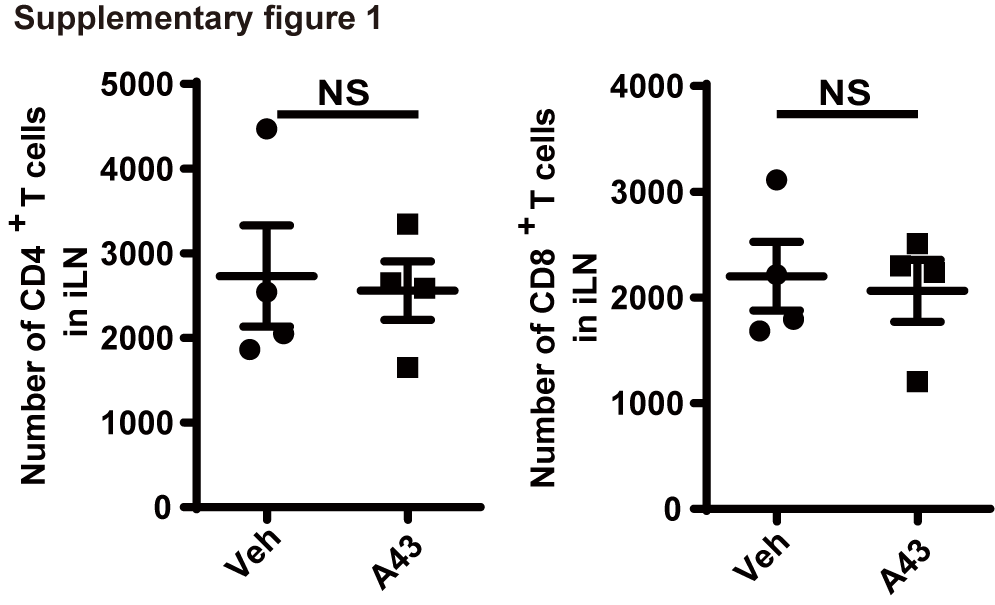

Supplement: Supplementary Figure 1 — P2X7R blockade does not affect T cell entry into LNs. 1 × 10^7^ CFSE-labeled lymphocytes were injected i.v. into recipients, and then A-438079 (200 μg/mouse) was administered i.p. Four hours later, the CFSE-labeled CD4+ or CD8+ naïve T cells in inguinal LNs were counted after gating on the CD49dlo cell fraction. Data represent the mean ± SEM of four mice. NS, not significant. [file Image_1.tif]

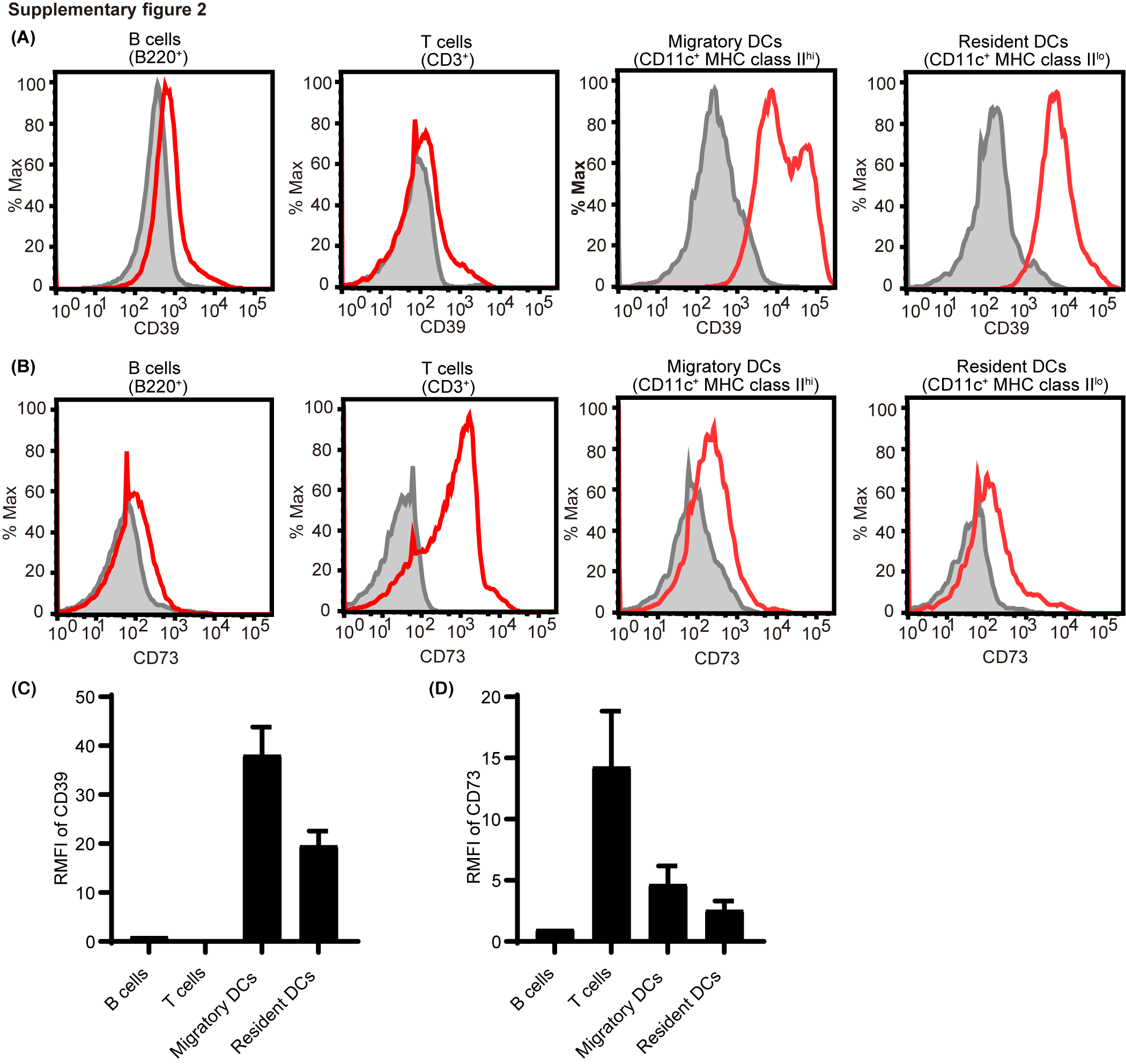

Supplement: Supplementary Figure 2 — Expression levels of CD39 and CD73 on T cells, B cells, and DCs. Expression of CD39 (A) and CD73 (B) on CD3+ T cells, B220+ B cells, CD11c+ MHC class IIhi migratory DCs, and CD11c+ MHC class IIlo resident DCs in LNs was analyzed by flow cytometry. CD39 and CD73 are shown in red. Isotype control is shown in grey. The histograms are representative of three independent experiments. Expression levels of CD39 (C) and CD73 (D) on B cells, T cells, migratory DCs, and resident DCs are comparatively shown. The RMFI indicates the relative median fluorescence intensities. Data represent the mean ± SD of three independent experiments. [file Image_2.tif]

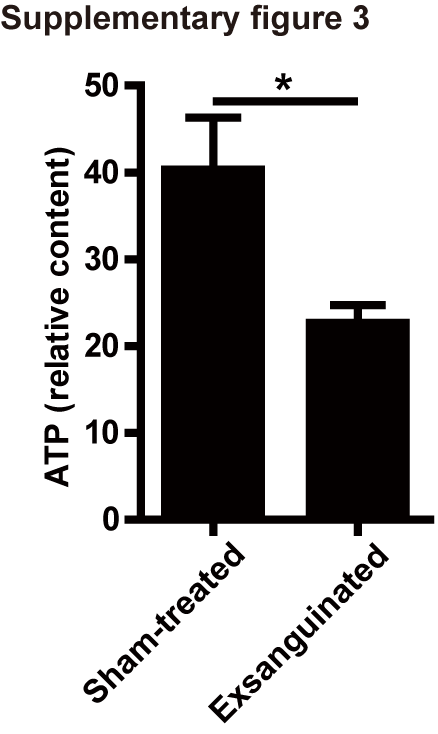

Supplement: Supplementary Figure 3 — Exsanguination reduces ATP levels in LNs. Mice were exsanguinated by puncture of the retro-orbital plexus under isoflurane anesthesia, and then inguinal LNs were collected. ATP contents in the LNs were determined by MALDI-IMS analysis. Data represent the mean ± SD of five or six replicates. *p<0.05 by Student’s t-test. [file Image_3.tif]

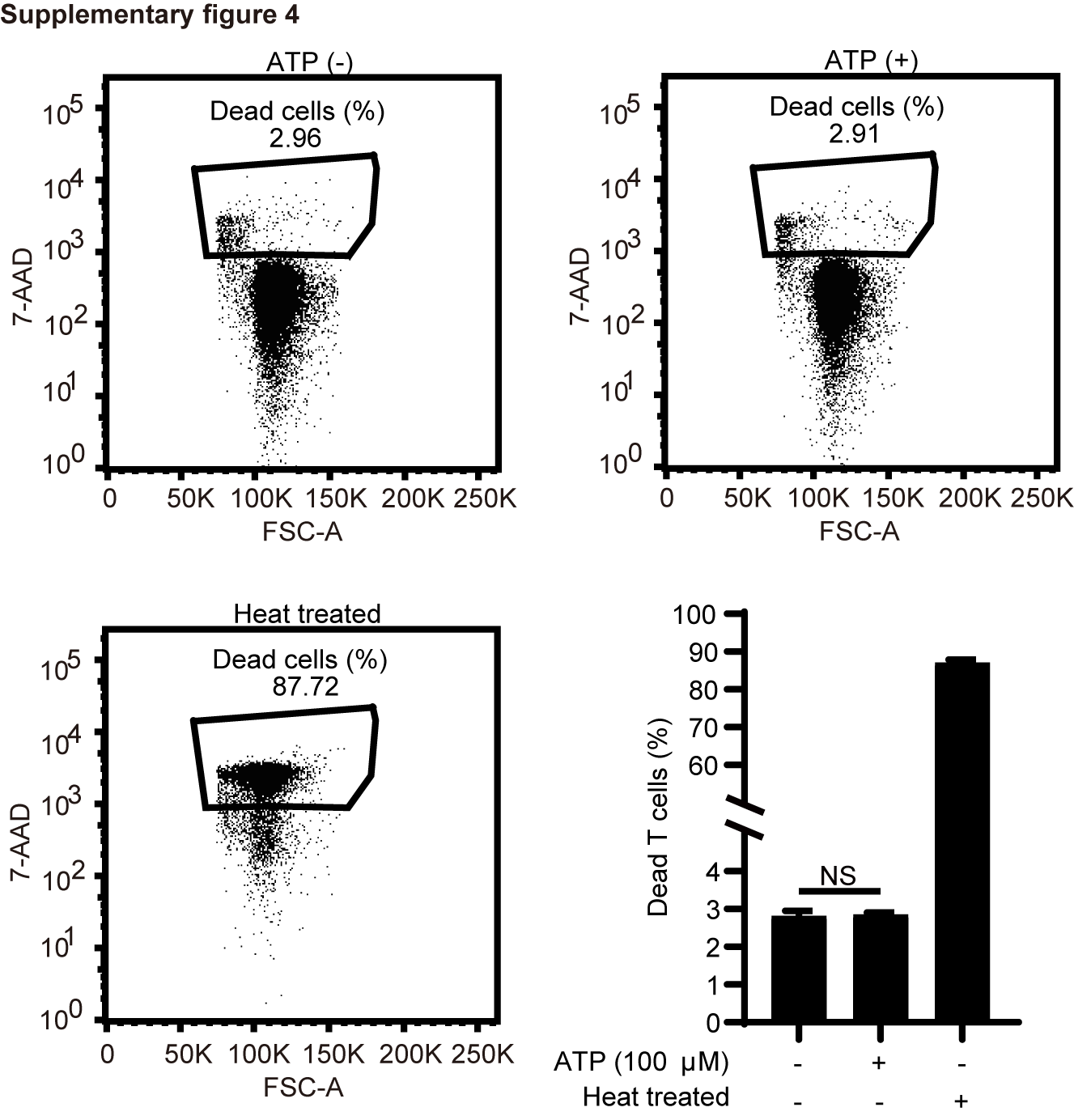

Supplement: Supplementary Figure 4 — The effect of ATP on T cell viability. LN cells were treated with or without 100 μM ATP. Two hours later, the frequency of 7-AAD+ CD4+ or CD8+ dead T cells was evaluated after gating on CD49dlo cell fraction. Heat-treated cells were used as a positive control. The results shown are representative of two independent experiments. Data represent the mean ± SD of triplicate wells. NS, not significant. [file Image_4.tif]

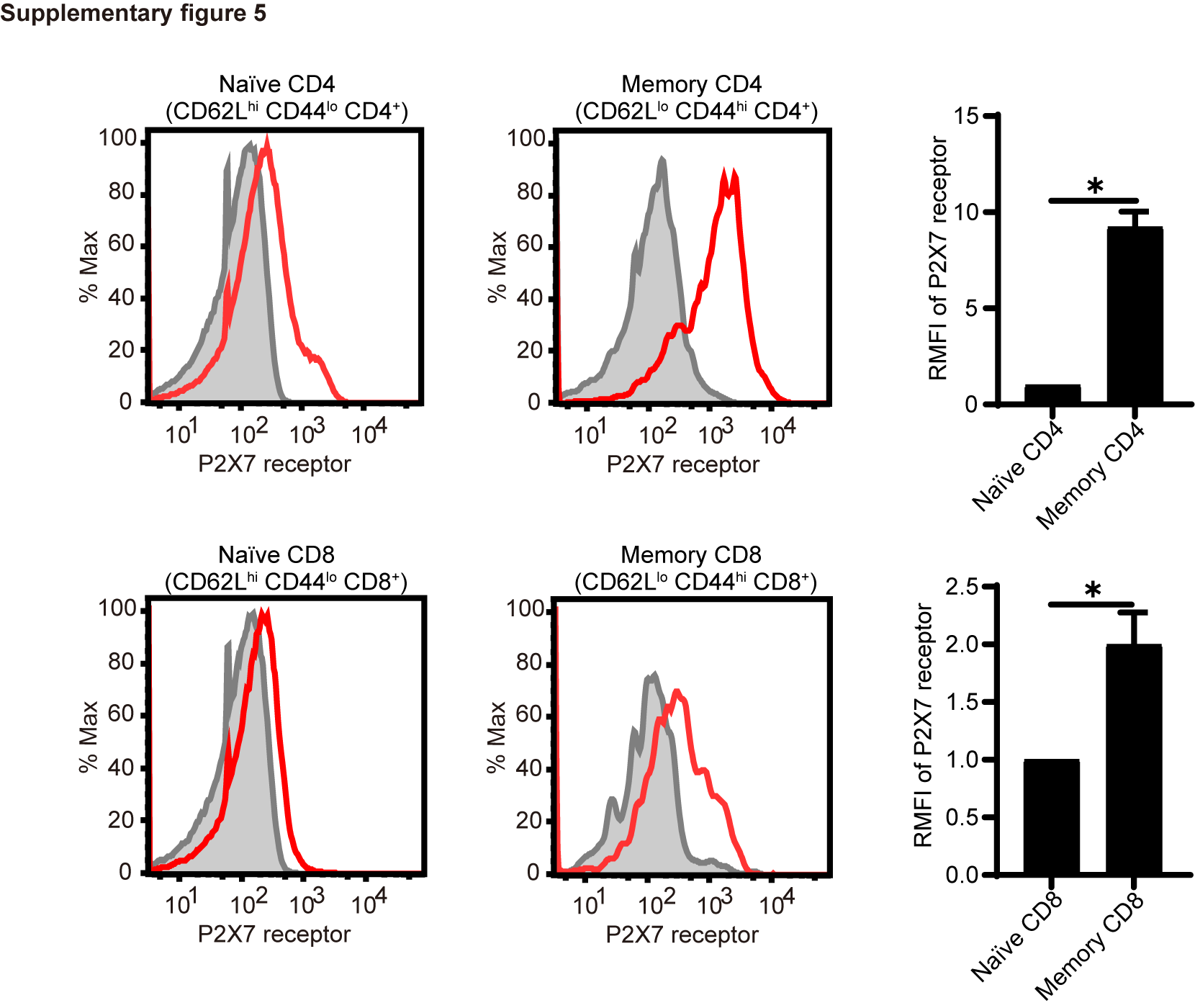

Supplement: Supplementary Figure 5 — P2X7 receptor expression on T cell substes in LNs. Expression of P2X7 receptor on CD62Lhi CD44lo naïve T cells (naïve) and CD62Llo CD44hi effector memory T cells (memory) in LNs was analyzed after gating on CD4+ or CD8+ cell fraction by flow cytometry. P2X7R is shown in red. Isotype control is shown in grey. The histograms are representative of three independent experiments. Bar graphs indicate RMFI of P2X7 receptor on naïve and memory T cells. Data represent the mean ± SD of three independent experiments. [file Image_5.tif]

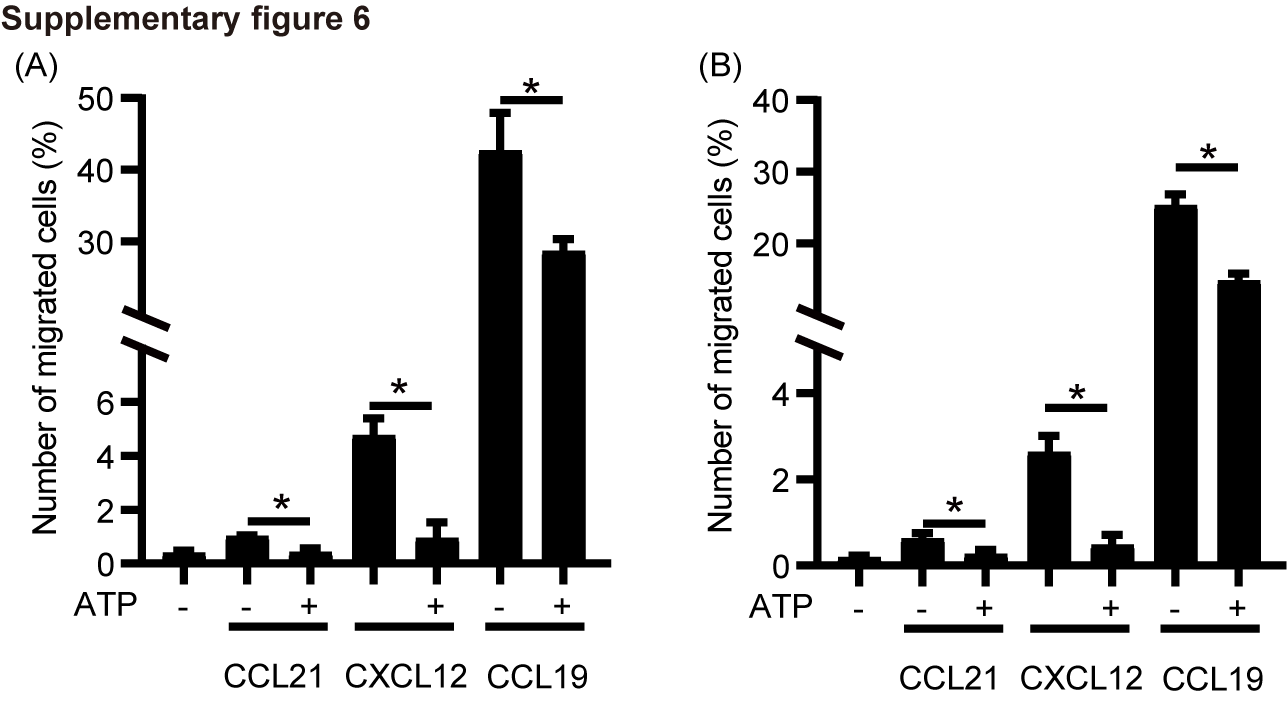

Supplement: Supplementary Figure 6 — ATP inhibits CXCL12, CCL19 and CCL21 dependent cell migration. T cell chemotaxis with or without ATP was analyzed by Transwell assay. (A) CD49dlo CD4+ T cells, (B) CD49dlo CD8+ T cells. The result shown is representative of two independent experiments. Data represent the mean ± SD of triplicate wells. *p<0.05 by Student’s t-test. [file Image_6.tif]

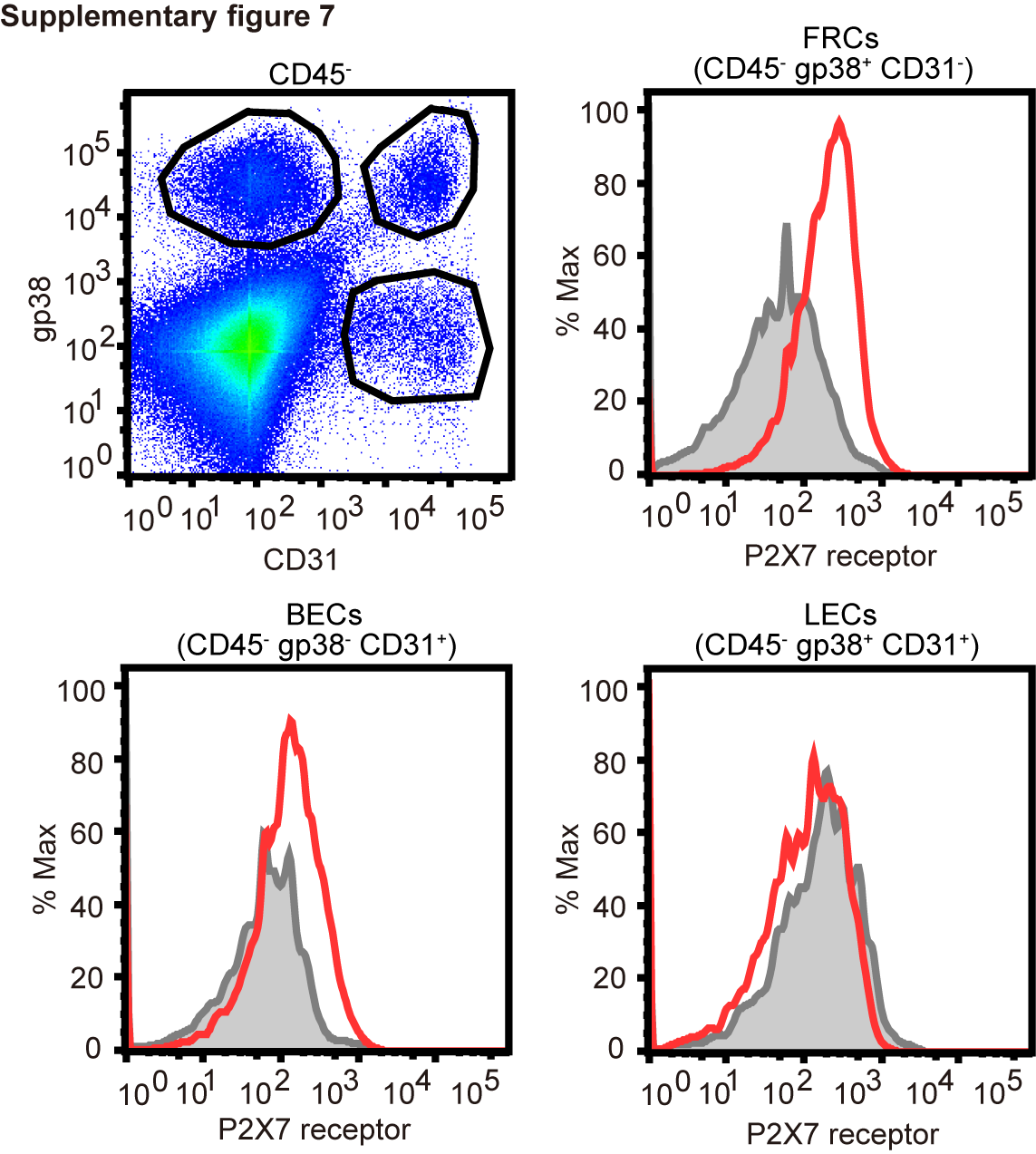

Supplement: Supplementary Figure 7 — P2X7R expression on stromal cells in LNs. Expression of P2X7R on CD45- gp38+ CD31- FRCs, CD45- gp38- CD31+ BECs and CD45- gp38+ CD31+ LECs in LNs was analyzed by flow cytometry. P2X7R is shown in red. Isotype control is shown in grey. The result shown is representative of two independent experiments. [file Image_7.tif]
